# Effect of a community-based intervention to increase participation in cervical cancer screening among Pakistani and Somali women in Norway

**DOI:** 10.1186/s12889-021-11319-1

**Published:** 2021-06-30

**Authors:** Samera Azeem Qureshi, Jannicke Igland, Kathy Møen, Abdi Gele, Bernadette Kumar, Esperanza Diaz

**Affiliations:** 1 Unit for Migration & Health, Norwegian Institute of Public Health (NIPH), P.O. Box 222, Skøyen, 0213 Oslo, Norway; 2 Department of Global Public Health and Primary Care, University of Bergen, P.O. Box 7804, N-5020 Bergen, Norway; 3 Department of Health and Caring Sciences, Western Norway University of Applied Sciences, Bergen, Norway; 4 The Norwegian Research Centre (NORCE) Alrek Helseklynge, Årstadveien 17, 5009 Bergen, Norway

**Keywords:** Immigrants, Community-based, Cervical Cancer, Screening, Evaluation

## Abstract

**Background:**

Norway implemented a regular cervical cancer screening program based on triennial screening in 1995, recommending participation of all women between 25 and 69 years of age. Somali and Pakistani women have the lowest participation in cervical cancer screening in Norway. This study evaluates the effect of a community-based intervention aimed at increasing participation in the screening program among women from these two groups.

**Methods:**

The intervention consisted of an oral 20–25 min presentation in Urdu and Somali on cervical cancer and screening and practical information on how to make an appointment and payment for the test. The participants were invited to pose questions related to the topic after the presentation. This study was carried out in four geographical areas surrounding the capital Oslo between February and October 2017, among women aged 25–69 years from Pakistan and Somalia. We recruited women in the intervention group directly from different community institutions, households, and religious sites. Women from Pakistan and Somalia residing in Oslo were the controls. The absolute intervention effect was measured as difference in absolute proportion of women screened and estimated as the interaction between time and group allocation in a generalized estimation equation model with binomial distribution and identity link function.

**Results:**

The percentage of women screened in the intervention group increased, from 46 to 51%. The corresponding increase in proportion in the control group was from 44 to 45.5%. After adjustment for potential confounders the intervention group showed a significant larger increase in participation in the screening program as compared to the control group with an absolute difference in change in proportion screened of 0.03 (95% CI; 0.02- 0.06).

**Conclusions:**

Our findings suggest that theory-based, culturally and linguistically sensitive educational interventions can raise awareness and motivate immigrant women to participate in cervical cancer screening program. In addition, approaching health professionals as well as immigrant women, might improve participation even more.

**Trial registration:**

NCT03155581. Retrospectively registered, on 16 May 2017.

**Supplementary Information:**

The online version contains supplementary material available at 10.1186/s12889-021-11319-1.

## Background

Little attention has been paid to immigrant health, especially, non-communicable diseases and cancer in particular [1]. There were an estimated 272 million international migrants in the world in 2019, which equates to 3.5% of the global population [2]. Immigration to the Nordic countries has increased considerably over recent decades and foreign-born individuals constitute a growing proportion of the local population. Norway is house According to Statistics Norway, 14.4% of total population are immigrants and 3.4% are Norwegians born to immigrant parents [3]. Somalis (28,642) are the third largest, whereas Pakistanis (20,647) are the fifth largest group of immigrants in Norway.

Cervical cancer is the second most commonly diagnosed cancer and the third leading cause of cancer deaths among females worldwide [4]. There were an estimated 570,000 new cervical cancer cases in 2018, making up almost 6.6% of all female cancer cases, and approximately 90% of the cancer deaths in low- and middle-income countries (LMIC) [5]. Globally, women from Sub-Saharan Africa and South-East Asia have the highest incidence of cervical cancer [6]. Substantial number of deaths due to cervical cancer can be prevented by screening and early detection. The absence of organized screening programs or low-uptake of screening contribute to the high prevalence of cervical cancer in LMIC [7, 8]. The low incidence and mortality among the majority populations in high-income countries could largely be due to organized screening and early detection [5].

In 1995, Norway implemented a regular cervical cancer screening (CCS) programme based on triennial screening. The Cancer Registry of Norway sends invitation letters in Norwegian to all women between 25 and 69 years of age. The occurrence of cervical cancer in Norway has been reduced by about 30% since the introduction of CCS in 1995 [9]. Immigrant women in general have low participation rate in CCS [10, 11], and Somali and Pakistani women have the lowest participation [10]. Published studies have also reported the barriers to uptake of CCS among immigrant women in Norway [12]. The low participation of immigrant women in screening could also partly be attributed to the health system and health providers, and not only to the women’s preferences. Møen *et.al.* describe health care providers biases, stereotypes and assumptions as one potential barrier to the low uptake among immigrant women [12] and a subsequent intervention targeting health care professionals has been effective [13]. Several types of interventions targeting individual immigrant women to increase their participation in CCS have been published [14–18]. However, to the best of our knowledge, no study has yet been published so far on the evaluation of a community-based intervention trial among immigrant women in Norway to increase participation in CCS.

In our community-based intervention, we have tried to target the whole Pakistani and Somali community rather than individual Pakistani and Somali women, and the involvement of the community will hopefully ensure the sustainability and impact of the intervention. This study evaluates the effect of this community-based intervention which was implemented among Somali and Pakistani women in Norway.

## Methods

Although the CONSORT guidelines for clinical trials (http://www.consort-statement.org/) are not applicable to our manuscript as described in detail below, we have tried to adhere to the checklist as much as possible. The detailed methodology and study design for the intervention has already been published and is described here briefly [19].

### Study design and randomization

This study was originally designed as a community-based cluster randomised intervention trial in four municipalities surrounding the capital Oslo (Lørenskog, Bærum, Asker and Drammen). Geographic areas within the municipalities were matched in pairs according to the number of Somali and Pakistani women living there and randomized as clusters. However, at the beginning of the intervention period we observed the presence of tight social bonds and common meeting places between the immigrants across the areas we had depicted. Immigrant women in the intervention group had family and friends in the control areas and sometimes women living in the control areas showed up to participate in meetings in the intervention areas. We realized that, in practice, discussion among the women about the intervention in control areas was unavoidable, resulting in a large risk of contamination of the control group. For this reason, we abandoned the cluster-randomized design and instead compared women living in the areas randomized to the intervention group against women living in Oslo. Oslo was chosen as control group because the city is geographically close to the four municipalities included in the trial and has similar demographics.

### Inclusion criteria

We included all women with immigrant background from Somalia or Pakistan (born in Somalia or Pakistan or Norwegian-born with Somali or Pakistani parents) who lived in the intervention areas or the control area as per January 1st 2017, were between 25 and 69 years of age January 1st 2017 and were registered as living in Norway throughout the whole period between January 1st 2012 and January 1st 2018.

### Sample size estimation and group allocation

For the original randomized study, the necessary sample size was calculated for an expected increase in cancer screening participation from 45 to 55%, 80% power and 5% significance level. At the time this study was developed, there was no available data on the percentage of immigrant women who participated in the CCS programme in Norway, and based on international studies [20, 21] and our own proxies [10], we conservatively estimated 45% screening participation in the control group. We obtained information on the number of Pakistani and Somali women aged 20–69 who lived in the four target municipalities from Statistics Norway [10]. When applying an average number of women of 612 per cluster (geographical areas with substantial female immigrant population) and an intra class correlation (ICC) of 0.025 in the sample size calculation, 32 clusters (16 intervention clusters and 16 control clusters) were enough. The 32 clusters from four different municipalities were matched in pairs according to the number of female immigrants aged 20 to 69 from Somalia and Pakistan and randomized pairwise. All Somali and Pakistani women who satisfied the inclusion criteria and were living in one of the 16 clusters randomized to receive the intervention were included in the intervention group (*n* = 1554). Because of the contamination described earlier we did not include women living in the 16 control clusters in the analyses as controls, but instead chose to use all Somali and Pakistani women living in Oslo as the control group (*n* = 9266). If the proportion screened is 45% in the control group, the minimal detectable difference in proportion screened with 80% power and 5% significance level with this sample size is 3.8%.

### Recruiting in the intervention areas

The principal author, a senior researcher of Pakistani origin, approached the Pakistani women by mobilizing different channels such as organizations and community centers to identify one informant in each of the four areas. With the use of personal social circle and involvement of organization and community centers, including the imams of the mosques in the intervention areas, four key informants were recruited, and they further recruited participants to a meeting in the area where they lived through different channels such as personal phone calls, community gatherings and social media (Facebook). Women who did not show up for the first meeting despite having agreed to it, were contacted again to attend a second meeting through personal visits, phone calls and through women who attended the first meeting. A Somali female research assistant recruited the Somali women adopting similar procedures. Among the 1123 Pakistani and 431 Somali women who were living in the intervention areas as indicated by The Statistics Norway (SSB) the key informants managed to invite a total of 401(36% of the Pakistani) and 378 (88% of Somali) women to one of the seven meetings arranged for each group. Among them, 102 (9% of the total and 25% of invited) Pakistani and 128 (30% of the total and 34% of invited) Somali women attended the meetings.

### Intervention

The intervention was carried out from February to October 2017. The details of the intervention are already published [19]. It consisted of a power-point presentation with a general description of our project aims followed by brief information on healthy lifestyle and preventative health care and then narrowing it down to cervical cancer. In very simple language (Somali or Urdu), we explained what cervical cancer is, its anatomical location -through diagrammatic illustration-, causes, risk factors, and development. This was followed by practical information on cervical cancer screening including the procedure and the instruments used, which were also shown physically. A short video clip of the test was played. In addition, the women were informed about the Norwegian screening guidelines for cervical cancer and on how to book an appointment with their GP for taking a screening test, including information about the payment. The content of the presentation (power point) was both in English and in the respective languages (Urdu and Somali).

### Outcome measure

For the evaluation of the intervention, we obtained data through three national registries, linked by the unique personal identification number available for each Norwegian resident.

Information on screening status at baseline and post-intervention was obtained from the Norwegian Cancer Registry where women between 25 and 69 years old are registered when they take the CCS test, provided they consent to registration. The main outcome measure was screening status as per 1st of January 2018. We defined a woman as screened if she had taken a CCS test within 3 years before January 1st, 2018. Likewise, a woman was defined as screened at baseline if she had taken a CCS test within 3 years before January 1st, 2017. A three-year time window was chosen since the recommendation in Norway is to take a CCS test every third year.

### Other covariates

Information about age per January 1st, 2017, years since immigration, country of origin, country of birth, marital status, income in 2016, and highest achieved level of education was obtained from Statistics Norway. Income was divided into quartiles based on the distribution of income for all Pakistani and Somali women aged 20–69 in Norway in 2016.

Information on the woman’s GP including GP’s age, gender and country of origin was obtained through linkage of data from the national GP database and Statistics Norway.

### Statistical methods

Baseline characteristics for the intervention group and the control group (Oslo) were compared using descriptive statistics with means and standard deviations for continuous variables and counts and percentages for categorical variables. Since the allocation of the intervention was not randomized between the two groups, we tested for baseline differences using t-tests for continuous variables and chi-square tests for categorical outcomes. We adjusted for education in a separate model as there were a lot of missing values for this variable. The possible reason could be that missing registration of education taken in the country of origin.

The effect of the intervention was estimated by applying generalized estimation equations (GEE) with binomial distribution and identity link function with screening status as the outcome using data in long format with two binary measurements of screening status per person (status January 1st 2017 and status January 1st 2018).

The effect of the intervention was obtained as the interaction term between a binary time-variable and the binary variable for intervention group in a model also containing a main effect for time and a main effect for intervention group. Effects on an absolute scale were obtained as the coefficient for the interaction term between time and intervention group and reported as risk differences (RD) with 95% confidence intervals. The confidence intervals were estimated with robust standard errors. The interaction effect can be interpreted as the difference in change in the absolute proportion screened with positive values indicating that screening participation has increased more in the intervention group compared to the control group.

We applied four levels of covariate adjustment; unadjusted (Model 1), adjustment for woman’s age, country of origin, first/second generation immigrant, marital status and income quartiles (Model 2), additional adjustment for GP’s age, GP’ gender and GP’ country of origin (Model 3) and finally additional adjustment for the woman’s educational level (Model 4). The adjustment variables were chosen because they have been found to be associated with participation in screening in previous studies [10, 11] and because they differed between the two groups at baseline.

As additional analysis we also applied propensity score stratification with deciles of propensity scores instead of traditional covariate adjustments. The propensity scores were obtained as predicted probabilities of being in the intervention group from a logistic regression model with intervention-group as the outcome and woman’s age, country of origin, marital status, income quartiles, GP’s age, GP’ gender and GP’ country of origin as independent variables.

As a second sensitivity analysis we applied the same GEE-models as described earlier to compare change in screening status from January 1st, 2016 to January 1st, 2017 between the intervention group and the control group. Since this was before the intervention, any differences would have to be caused by something else than the intervention.

We also did subgroup analyses to compare the strength of the effect of the intervention between Somali women and Pakistani women and between first and 2nd generation immigrants. Effect modification by country of origin and by generation was evaluated by including a 3-way interaction between intervention group, time and country of origin in GEE models.

All tests are two-sided, and 5% significance level was applied in all analyses. All analyses were conducted using Stata MP version 16.

#### Ethics

The trial is registered in ClinicalTrials.gov with Protocol ID: 2015/1156 and identifier NCT03155581. All the data included in the current paper are obtained from national registers. Women’s cervical cancer status is only registered in the national register if they have given consent. Our project obtained ethics approval from the Regional Ethics Committee of Norway (REK), number 2015/1156/REK Vest, including exception for consent for the data collected from registers.

## Results

Our study included 10,820 immigrant women, 1554 (14.4%) living in the intervention areas and 9266 (85.6%) in the control areas. Table 1 describes the baseline characteristics of both the intervention and the control groups. Compared to the control group the intervention group had a larger proportion of Pakistani, married women with higher education and income. They also had a higher proportion of GPs who were female, older and of Norwegian origin. Baseline characteristics further stratified by country of origin (Pakistan and Somalia) are reported in Supplementary Table 1.

**Table 1:** Demographic characteristics of participants in intervention and control groups

Intervention group	Intervention group	Control group	***P***-value
**n**	1554	9266	
**Age, mean (SD)**	41.2 (11.3)	40.9 (11.2)	0.27
**Born in Norway, n (%)**	371 (23.9)	1838 (19.8)	< 0.001
**Age when arrived in Norway**^a^**, mean (SD)**	21.2 (9.6)	21.3 (9.4)	0.85
**Years since arrived in Norway**^a^**, mean (SD)**	22.4 (10.1)	22.2 (11.0)	0.58
**Country of origin, n (%)**			
Somalia	431 (27.7)	2958 (31.9)	
Pakistan	1123 (72.3)	6308 (68.1)	0.001
**Marital status, n (%)**			
Unmarried	259 (16.7)	1808 (19.5)	
Married/Partner	1009 (64.9)	5435 (58.7)	
Divorced/Separated	245 (15.8)	1701 (18.4)	
Widow	41 (2.6)	322 (3.5)	< 0.001
**Education, n (%)**			
Primary	606 (39.0)	4013 (43.3)	
High school or vocational school	309 (19.9)	1881 (20.3)	
College/University	406 (26.1)	1776 (19.2)	
Missing	233 (15.0)	1596 (17.2)	< 0.001
**Income quartile in 2016**			
Q1: (0–50,000 NOK)	257 (16.5)	2319 (25.0)	
Q2: (50,000 NOK - 420,000 NOK)	337 (21.7)	2128 (23.0)	
Q3: (420,000 NOK – 770,000 NOK)	449 (28.9)	2443 (26.4)	
Q4: > 770,000 NOK	511 (32.9)	2376 (25.6)	< 0.001
**Female GP, n (%)**	1037 (66.9)	5713 (61.7)	< 0.001
**Age of GP, n (%)**	50.6 (9.7)	49.6 (10.0)	< 0.001
**Country of origin for GP, n (%)**			
Norway	948 (61.2)	3632 (39.2)	
European country	228 (14.7)	1550 (16.8)	
Non-European country	374 (24.1)	4073 (44.0)	< 0.001
**Screened at least once the last 4 years, n (%)**	713 (45.9)	4088 (44.1)	0.2

^a^Only calculated for those who were not born in Norway

Table 2 shows the proportion of women screened at baseline and follow-up in the intervention and the control groups and the intervention effect estimated using GEE models. The crude percentage of women screened in the intervention group increased to 50.8% as compared to 45.9% at baseline, an absolute increase of 4.9%. The corresponding increase in percentage in the control group was from 44.1 to 45.5%. The unadjusted intervention effect was 0.03 and statistically significant (95% CI 0.01–0.06), indicating that the absolute change in percentage screened was 3 % points higher in the intervention group. The effect did not change much after successive adjustment for covariates in model 2–4 and was still significant. In additional analyses using propensity score stratification instead of covariate adjustment, we obtained an absolute effect estimate of 0.05 with 95% CI 0.02–0.07.

**Table 2:** Effect of the intervention on change in cervical cancer screening (CCS) participation among immigrant women

	Screened January 2017, n (%)		Screened January 2018, n (%)		Absolute difference in change in proportion screened^e^ B (95%CI)
	Control group	Int. group	Control group	Int. group	
Total sample					
Model 1^a^	4088/9266 (44.1)	713/1554 (45.9)	4219/9266 (45.5)	789/1554 (50.8)	0.03 (0.01-0.06)
Model 2^b^	4088/9266 (44.1)	713/1554 (45.9)	4219/9266 (45.5)	789/1554 (50.8)	0.04 (0.01-0.06)
Model 3^c^	4083/9255 (44.1)	711/1550 (45.9)	4216/9255 (45.6)	789/1550 (50.9)	0.04 (0.02-0.06)
Model 4^d^	3469/7663 (45.3)	626/1318 (47.5)	3584/7663 (46.8)	685/1318 (52.0)	0.03 (0.01-0.05)

^a^Adjusted for baseline screening status

^b^Adjusted for covariates in model 1 plus additional adjustment for age, country of origin, first/second generation immigrant, marital status and income quartiles

^c^Adjusted for covariates in model 2 plus additional adjustment for GP’s gender, GP’s age and GP’ country of origin

^d^Adjusted for covariates in model 3 plus additional adjustment for level of education (reduced sample size because of missing values)

^e^ Absolute difference in change in screening participation estimated as interaction between year and intervention group in generalized estimating equation model (GEE) with binomial distribution and identity link function

When estimating difference in change in screening participation in the period January 1st 2016 to January 1st 2017 there was no significant difference between the intervention group and the control group (Supplemental Table 2) and the estimate of the difference was close to zero (0.01, 95% CI: − 0.02, 0.03).

Table 3 shows subgroup analysis by country of origin and by immigrant generation. The estimate of intervention effect was stronger in Somali women compared to Pakistani women and failed to reach significance among Pakistani women for all four models. The test for effect-modification by country of origin through a 3-way interaction between intervention group, time and country of origin was not significant for any of the four levels of covariate adjustments (*p*- value for interaction terms 0.12, 0.15, 0.12 and 0.06 in model 1, 2 3 and 4 respectively). For analyses stratified on immigrant generation we found a significant effect of the intervention among 1st generation immigrants, but among 2nd generation immigrants there was no effect of the intervention. The 3-way interaction between intervention group, time and immigrant generation was however not significant (*p*-value for interaction term 0.06, 0.054, 0.06 and 0.13 in model 1, 2 3 and 4 respectively).

**Table 3:** Effect of the intervention on change in participation in cervical cancer screening (CCS) in sub-groups of immigrant women

	Screened January 2017, n screened/n total (%)		Screened January 2018, n screened/n total (%)		Absolute difference in change in proportion screened^e^ (95%CI)
	Control group	Int. group	Control group	Int. group	
**By country of origin**					
**Somali**					
Model 1^a^	1248/2958 (42.2)	172/431 (39.9)	1278/2958 (43.2)	203/431 (47.1)	0.06 (0.02–0.10)
Model 2^b^	1248/2958 (42.2)	172/431 (39.9)	1278/2958 (43.2)	203/431 (47.1)	0.06 (0.02–0.10)
Model 3^c^	1245/2952 (42.2)	170/429 (39.6)	1270/2952 (43.3)	203/429 (47.3)	0.07 (0.03–0.11)
Model 4^d^	1002/2306 (43.5)	143/350 (40.9)	1026/2306 (44.5)	170/350 (48.6)	0.07 (0.03–0.11)
**Pakistani**					
Model 1^a^	2840/6308 (45.0)	541/1123 (48.2)	2941/6308 (46.6)	586/1123 (52.2)	0.02 (−0.00–0.05)
Model 2^b^	2840/6308 (45.0)	541/1123 (48.2)	2941/6308 (46.6)	586/1123 (52.2)	0.02 (−0.00–0.05)
Model 3^c^	2838/6303 (45.0)	541/1121 (48.3)	2938/6303 (46.6)	586 /1121 (52.3)	0.02 (−0.00–0.05)
Model 4^d^	2467/5357 (46.1)	483/968 (49.9)	2558/5357 (47.8)	515/968 (53.2)	0.02 (−0.00–0.04)
**By immigrant generation**					
**1st generation**					
Model 1^a^	3400/7428 (45.8)	536/1183 (45.3)	3429/7428 (46.2)	593/1183 (50.1)	0.04 (0.02–0.07)
Model 2^b^	3400/7428 (45.8)	536/1183 (45.3)	3429/7428 (46.2)	593/1183 (50.1)	0.04 (0.02–0.07)
Model 3^c^	3395/7417 (45.8)	534/1180 (45.3)	3426/7417 (46.2)	593/1180 (50.3)	0.05 (0.02–0.07)
Model 4^d^	2794/5872 (47.6)	451/956 (47.2)	2807/5872 (47.8)	490/956 (51.3)	0.04 (0.01–0.07)
**2nd generation**					
Model 1^a^	688/1838 (37.4)	177/371 (47.7)	790/1838 (43.0)	196/371 (52.8)	−0.00 (−0.05–0.04)
Model 2^b^	688/1838 (37.4)	177/371 (47.7)	790/1838 (43.0)	196/371 (52.8)	−0.00 (− 0.05–0.04)
Model 3^c^	688/1838 (37.4)	177/370 (47.8)	790/1838 (43.0	196/370 (53.0)	−0.00 (− 0.05–0.04)
Model 4^d^	675/1791 (37.7)	175/362 (48.3)	777/1791 (43.4)	195/362 (53.9)	−0.00 (− 0.05–0.04)

^a^Adjusted for baseline screening status

^b^Adjusted for covariates in model 1 plus additional adjustment for age, country of origin or first/second generation immigrant, marital status and income quartiles

^c^Adjusted for covariates in model 2 plus additional adjustment for GP’s gender, GP’s age and GP’ country of origin

^d^Adjusted for covariates in model 3 plus additional adjustment for level of education (reduced sample size because of missing values)

^e^ Absolute difference in change in screening participation estimated as interaction between year and intervention group in generalized estimating equation model (GEE) with binomial distribution and identity link function

## Discussion

This community-based intervention study among Somali and Pakistani women in four counties in Norway shows a significant larger increase in the uptake of cervical cancer screening test among the immigrant women in the intervention group compared to the control group. In subgroup analyses we found that this effect was mostly restricted to Somali women and 1st generation immigrants.

Second generation women are per definition born in Norway and speak fluent Norwegian. In addition, they have attended Norwegian school and are more acculturated than their mothers. Although previous studies show that not all immigrant groups change their use of prevention as quickly while living in Norway [22], it is as expected that a targeted intervention is not so useful in this group. One plausible explanation for less effect among Pakistani women can be that they were to a much greater extent 2nd generation immigrants. And among those who were 1st generation immigrants, the Pakistanis had lived here longer. The Pakistanis women were more educated, married, and had higher income. Table 3 also shows Pakistanis were to a greater extent “screened” at baseline in the intervention group. They therefore had less potential for improvement than Somali women.

Our findings are similar to those reported by a number of meta-analyses and systematic reviews based on studies from different countries stating that culturally tailored interventions targeting individual minority women can increase attendance to mammographic and cervical cancer screening [14, 15, 17]. Brevik *et.al* in their meta-analysis describe the effect of an educational intervention that resulted in a 54% increase at the Pap test (RR = 1.54, 95% CI, 1.14–2.09, *P* = .005) [14]. However, all the studies included in the review were from the USA, and the intervention targeted individual women in contrast to our intervention which targeted a community. Another review study also concluded that health education models (postcards, brochures, power point slides, videos etc.) are helpful in increasing participation in cancer screening [23].

Our intervention was community-based, where the word of mouth effect also plays an important role. Women who attended the meeting were encouraged to discuss about the intervention with the women who were not able to attend. The study populations were oral communities in which the word of mouth spreads very quickly and the *diffusion of information* occur through social networks, which is a prominent tool for information sharing *among Somali and Pakistani first generation immigrants.* Therefore, although not attending the intervention meetings, the information can reach other women as well. Furthermore, previously published studies have highlighted the methodological challenges of recruiting minority groups into research trials [24–27]. Thus, although fewer women than invited attended the meeting but the information did reach many. At the same time previously published studies have highlighted the methodological challenges of recruiting minority groups into research trials [24–27].

In addition, the effect size of the intervention seen among Somali women was a very positive feedback. One possible explanation for more effectiveness in Somali women could be more frequent interaction between Somali women than Pakistani women which helped spread the information about the benefits of screening. Basically, the Somali community is a close community where women share their experiences and encounters with their families and friends more frequently. Language barrier has been reported by many studies as one important reason among Somali and other immigrant women in general for not participating in cervical cancer screening [7, 28].

However, even though we observe a significant effect of the intervention, our results should be interpreted with caution. As we had to change the study design, the lack of randomization might have precluded us from controlling for all confounding factors and our results might have been affected by residual confounding. However, we did try to control for most of the obvious confounding factors that have been found to be associated with screening participation in previous studies [29]. Also, when we analyzed changes in screening participation the year before the intervention, we found no difference in change between the intervention group and the control group. We assume that if the differences we observed between the groups the year of the intervention were caused by confounding factors, there should also be differences between the groups the previous year. The lack of such differences supports our finding of a significant intervention effect.

Another study, conducted by the same research group, on the evaluation of an intervention among health care providers also showed a significant effect (absolute effect size 2%) and increased the participation in screening among immigrant women [13]. However, when we directed the intervention to the women as opposed to health providers, the effect size increased to 5%. The results from these two interventions imply that lower attendance to CCS by immigrants could be increased by interventions both among the women themselves and also by targeting the health care providers in the host country. Even though the effect sizes are small, the clinical impact of these interventions might be of importance for the individual woman, as Somali and Pakistani women are otherwise hard to reach. The degree to which these two interventions, one targeting health care providers and the other among immigrant women, can complement each other, should be further investigated.

Published literature identifies successful community-based interventions carried out among immigrant groups in other parts of the world [16, 18, 30–34]. These interventions included predominantly the use of community lay-health workers, linguistically appropriate and culturally tailored educational materials, and navigation assistance to overcome the barriers to access the services. These interventions have resulted in increase in awareness and knowledge about cervical cancer with increase in screening participation [16]. In their article on development of culturally adapted interventions Terragni *et.al.* highlighted four different themes such as involvement of target population at a very early stage- in order to gain their trust; enough time for development of the intervention and then execution of the main intervention; consideration of heterogeneity among different immigrant groups and finally actively working to gain support of the public health authorities thus facilitating the cultural adaptation and sustainability of initiatives reference. Our intervention was a culturally adapted intervention in many respects, including early involvement of the two communities through focus group discussions [28], selection of language, gender, venue and development of material [19] thus encompassing heterogeneity.

### Strengths and limitations

This is the first of its kind study to evaluate the effect of a community-based intervention among immigrant women in Norway. One of the key strengths of this study was the measurement of cancer screening using registry data rather than self-report, which tends to overestimate screening and varies with ethnicity [35, 36].

The most important limitation was that we had to change the initial design of cluster randomization because of possible contamination. We are aware that, the lack of randomization poses challenges such as selection bias and confounding. Which not only questions the validity and usefulness of the findings, but in addition non-randomized design is associated with a high risk of bias because known and unknown characteristics may be distributed unequally between groups [37]. Same may be the case with our findings, we cannot rule out that the observed effect is at least partly caused by residual confounding. In addition, contamination between the two groups may have decreased the effect size. However, all analyses conducted to explore this possibility point to a real effect of the intervention. Previously published studies have highlighted the methodological challenges of recruiting minority groups into research trials [24–27]. Another limitation worth mentioning is, we did not include men as targets of intervention in our study. Health behavior changes of an individual are complex processes where the individual, her surrounding family, health care providers and the health system mediators take part to achieve a positive outcome. In cultures where males have the power of decision in regarding the participation of women in the screening program it might have been positive to include the male members of the Somali and Pakistani communities and give them information on CCS and why it is necessary [32, 33].

Our study showed increased CCS after attendance to one short meeting with educational information, the effect of which was measured within 1 year after the intervention. Thus, we are unable to comment on the long-term effect of the intervention which may not be visible in a short time in fact the effect might not be sustainable without reminders. Unfortunately, our study material did not allow to have a longer study period.

### Implications for future research

Cost-effective analyses of both the interventions separately, the intervention among immigrant women and the intervention among GP done previously by the research team, as well as a study including both interventions simultaneously and over a longer time period, would help shaping and adaption of the right policy. Our findings also suggest that we need comprehensive measures directed at increasing participation in screening and preventing cervical cancer among immigrant women.

## Conclusions

Our findings suggest that a community-based educational intervention among Somali and Pakistani women can raise awareness and motivate immigrant women to participate in cervical cancer screening program.

## Supplementary Information

**Additional file 1: Supplemental Table 1.** Baseline characteristics for the study participants.

## Data Availability

The data used in this article is entirely from the Population Registry (https://www.skatteetaten.no/en/person/national-registry/), the Cancer registry (https://www.kreftregisteret.no/en/) and National GP database (https://www.helsenorge.no/fastlegen/om/) and Statistics Norway (https://www.ssb.no/en), which we are not allowed to share. Our project obtained ethics approval from the Regional Ethics Committee of Norway (REK), number 2015/1156/REK Vest for use of the data.

## Abbreviations

CCS: Cervical Cancer Screening
GP: General Physician
GEE: Generalized estimating equation model
LMIC: Low- and Middle-Income Countries
SSB: Statistics Norway
