# Supplementary material for: Effect of a community-based intervention to increase participation in cervical cancer screening among Pakistani and Somali women in Norway
Source: BMC Public Health. 2021 Jun 30;21:1271. doi: 10.1186/s12889-021-11319-1 (PMC8243573; doi:10.1186/s12889-021-11319-1)
Supplement: Supplementary file 1 — Additional file 1: Supplemental Table 1. Baseline characteristics for the study participants. [file 12889_2021_11319_MOESM1_ESM.docx]

***Supplementary Table 1: Baseline characeristics for the study participants***

|  | **Intervention** | | **Control** | |
| --- | --- | --- | --- | --- |
|  | **Somali n (%)** | **Pakistani (%)** | **Somali n (%)** | **Pakistani n (%)** |
| **Number of women** | 431 | 1123 | 2958 | 6308 |
| **Age mean (SD)** | 39.2 (9.6) | 41.6 (11.7) | 39.5 (9.7) | 42.1 (11.9) |
| **Age when arrived in Norway*, mean (SD)** | 23.8 (9.0) | 19.9 (9.3) | 23.0 (9.4) | 20.1 (9.5) |
| **Years since arrived in Norway*, mean (SD)** | 15.7 (6.2) | 25.8 (11.4) | 16.8 (6.3) | 25.9 (10.4) |
| **Born in Norway, n (%)** | 11 (2.6) | 360 (32.1) | 64 (2.2) | 1774 (28.1) |
| **Marital status** |  |  |  |  |
| Unmarried | 128 (29.7) | 131 (11.7) | 958 (32.4) | 850 (13.5) |
| Married | 152 (35.3) | 857 (76.3) | 871 (29.5) | 4564 (72.4) |
| Separated/Divorced | 141 (32.7) | 104 (9.3) | 1021 (34.5) | 680 (10.8) |
| Widow | 10 (2.3) | 31 (2.8) | 108 (3.7) | 214 (3.4) |
| **Education** |  |  |  |  |
| Primary education | 188 (43.6) | 418 (37.2) | 1485 (50.2) | 2538 (40.1) |
| High school  or vocational school | 95 (22.0) | 214 (19.1) | 504 (17.0) | 1377 (21.8) |
| University/college | 68 (15.8) | 338 (30.1) | 321 (10.9) | 1455 (23.1) |
| Missing | 80 (18.6) | 153 (13.6) | 648 (21.9) | 948 (15.0) |
| **Quartiles of income in NOK, n (%)** |  |  |  |  |
| Q1(0 - 50000 NOK) | 124 (28.8) | 133 (11.8) | 1405 (47.5) | 914 (14.5) |
| Q2 (50000 NOK - 420 000 NOK) | 139 (32.3) | 198 (17.6) | 810 (27.4) | 1318 (20.9) |
| Q3 (420 000 NOK – 770 000 NOK) | 102 (23.7) | 347 (30.9) | 497 (16.8) | 1946 (13.9) |
| Q4 >770 000 NOK | 66 (15.3) | 445 (39.6) | 246 (8.3) | 2130 (30.8) |
| **Female GP, n (%)** | 253 (59.0) | 784 (69.9) | 1347 (45.6) | 4366 (69.3) |
| **Age of GP, n (%)** | 49.6 (9.5) | 51.0 (9.8) | 49.9 (10.2) | 49.5 (9.9) |
| **Country of origin for GP, n (%)** |  |  |  |  |
| Norway | 273 (63.6) | 675 (60.2) | 1169 (39.6) | 2463 (39.1) |
| European country | 80 (18.7) | 148 (13.2) | 494 (16.7) | 1056 (16.8) |
| Non-European country | 76 (17.7) | 298 (26.6) | 1289 (43.7) | 2784 (44.2) |

***Supplementary Table 2: Estimates of difference in change in screening participation between the intervention group and the control group the year before the intervention***

|  | **Screened**  **January 2016, n (%)** | | **Screened**  **January 2017, n (%)** | | **Absolute difference in change in proportion screened****  **B (95 %CI)** |
| --- | --- | --- | --- | --- | --- |
|  | **Control group** | **Int. group** | **Control group** | **Int. group** |  |
| **Total sample** |  |  |  |  |  |
| **Model 1^1^** | 3965/9266 (42.8) | 708/1554 (45.6) | 4088/9266 (44.1) | 713/1554 (45.9) | -0.01 (-0.03, 0.01) |
| **Model 2^2^** | 3965/9266 (42.8) | 708/1554 (45.6) | 4088/9266 (44.1) | 713/1554 (45.9) | -0.01 (-0.03, 0.01) |
| **Model 3^3^** | 3960/9255 (42.8) | 706/1550 (45.6) | 4083/9255 (44.1) | 711/1550 (45.9) | -0.01 (-0.03, 0.01) |
| **Model 4^4^** | 3370/7663 (44.0) | 600/1318 (45.5) | 3469/7663 (45.3) | 626/1318 (47.5) | 0.01 (-0.02, 0.03) |
